# Aeromedical evacuations during the COVID-19 pandemic: practical considerations for patient transport

**DOI:** 10.1017/cem.2020.434

**Published:** 2020-06-24

**Authors:** Francois Lemay, Abel Vanderschuren, Judith Alain

**Affiliations:** * Département d'anesthésiologie, CHU de Québec Université Laval, Hôtel-Dieu de Québec, Québec, QC; † Service d’évacuations aéromédicales du Québec, CHU de Québec Université Laval, Québec, QC; ‡ Département de médecine, CHU de Québec Université Laval, Québec, QC; § Département de médecine d'urgence. CHU de Québec Université Laval, Québec, QC

**Keywords:** aeromedical, COVID-19, transport

Aeromedical evacuation systems around the world face new challenges in light of coronavirus disease 2019 (COVID-19).^1^ These challenges include unprecedented demand for patient transfers, as well as increased risk of exposure to aircraft crew due to prolonged close contact with contagious patients. Our organization, Service d’évacuations aéromédicales du Québec (EVAQ, Quebec Aeromedical Evacuation Services) is the medical evacuation service for the Province of Quebec. Every year, over 2000 critical care patients are transferred by our service. Since the declaration of the COVID-19 pandemic by the World Health Organization on March 11, 2020, EVAQ has organized transfers for 50 COVID-19 confirmed or suspected patients. We would like to share some practical considerations from our experience with aeromedical transfer of COVID-19 patients. These concepts are relevant not only to aeromedical transfer crews, but also to the referring and receiving emergency medical teams.

1. Early endotracheal intubation has an increased role in suspected COVID-19 patients. Preemptive intubation is already a strategy being recommended for any patient with the potential of respiratory decompensation during aeromedical transport, regardless of COVID-19 status.^2^ Throughout the pandemic, medical societies have been advocating for early intubation to manage respiratory distress.^3^ This recommendation stems from efforts to reduce the use of noninvasive ventilation (NIV), which is linked to increased transmission of respiratory infections.^4^ Early intubation can impact on centers where transfers are made before the patient deteriorates and needs intubation. Referring centers must be aware of this potential change in their usual practice and could have to intubate more frequently patients before transport. We recommend that collaborating facilities and aeromedical organizations develop and share their protocols regarding their approach to NIV, tracheal intubation, and ventilator circuit exchange procedure for of mechanically ventilated COVID-19 patient. This will increase efficiency of transfers and decrease risks of errors.
2. There is a potential benefit to prone positioning in COVID-19 patients with acute respiratory distress syndrome (ARDS).^5^ Sending and receiving facilities must be aware if their local aeromedical evacuation system can perform proning. Boarding a patient carries a risk for accidental dislodgement of equipment, such as endotracheal tubes and intravenous accesses, and this could have dramatic consequences in proned patients. Turning the patient during flight, in any emergent situation, is impossible in many clinical settings. We believe that patients with ARDS should be transferred early, before facing refractory hypoxemia. This could avoid the risks of needing advanced respiratory management strategies during patient transfer.
3. Communication in all steps of patient transport is difficult with COVID-19 patients. Before transport, instructions must be transmitted before performing transfers so that local teams can adequately prepare the patient. During transfer and boarding, loud noise from the aircraft is a major barrier to verbal communication. Personal protective equipment (PPE), especially masks and visors, increases this difficulty. Moreover, equipment such as cell phones cannot be held in proximity to the medical staff's head to limit contamination. The use of speaker modes of any communication devices, because of background noise, is impossible. Thus, informing receiving teams of acute changes in the patient's condition before arrival is limited. To address these limitations, comprehensive information on patients’ condition must be communicated before arrival of the aeromedical team to any location. During handover of the patient to the aeromedical team, communication should focus on acute changes since previous communications. Finally, dedicated medical personnel who remain in an uncontaminated zone of the aircraft can provide receiving facilities with information on changes in patient condition during flight.
4. Time to complete a given transfer will be longer than previously necessary. Patient care will be prolonged due to increased complexity of patient preparation and PPE. Operational changes also have a great impact on transfer efficiency. For example, additional time is required in between transfers for decontamination of the aircraft, which takes several hours. Furthermore, staff must be protected at all times from potential emergencies associated with high-altitude flights. For example, if cabin decompression occurs, passengers will be required to put on oxygen masks. If this cannot be safely done over the PPE, this could involve removing the PPE, such as N95 masks, thereby exposing staff to viral particles. To eliminate this risk, our organization has opted to fly at a lower altitude, which increases flight duration for any given route. Thus, sending facilities might have to stabilize patients for longer periods, and receiving ones may expect longer delays.
5. Limiting cross-contamination of the crew is of utmost importance and represents a challenge in the close quarters of an aircraft. In our organization, all on-board members must wear a procedural mask at all time when flying due to their proximity, and switch to a N95 mask immediately before the patient is taken onto the aircraft. Aeromedical organizations, depending on their aircraft and staff, will also have new, specific procedures to limit cross-contamination. For example, the DHC-8 airplanes used by our organization are larger than average aeromedical aircraft, which allows for separation of a “hot” zone (contaminated area with patients), a transition zone and a “cold” zone (uncontaminated area for crew). This type of airplane is different from the smaller Challenger airplanes that we usually use for such critical care patients. This change increases flight duration and modifies boarding procedure. Figure 1 demonstrates the DHC-8 aircraft with explanations of the zones.

**Figure 1.**
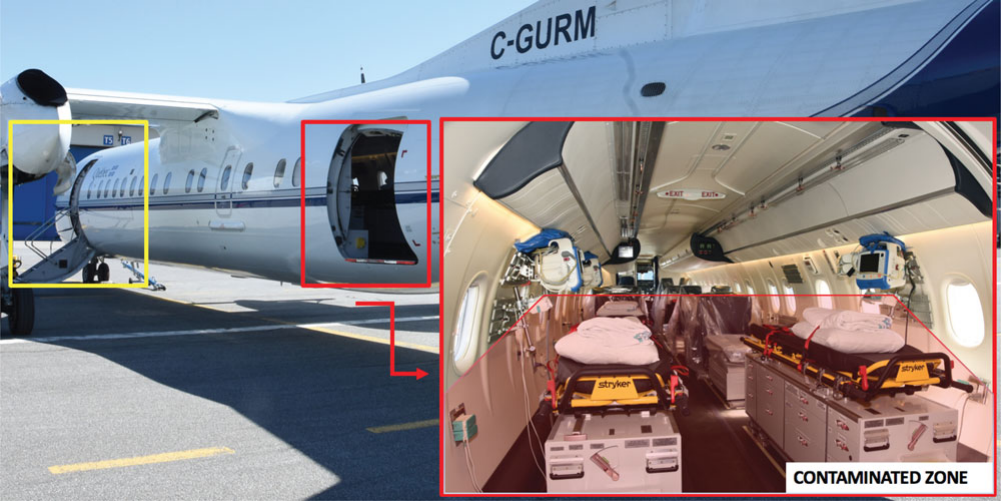
Representation of boarding zones for the aircraft, whereby uncontaminated personnel board through the front door (yellow), and patients and treating medical staff board through the cargo door (red). This allows for safe transition zones within the airplane (De Havilland Canada DHC-8, Service d’évacuation aéromédicales du Québec).

We hope that our experience will provide insight for emergency and critical care professionals involved with interfacility patient transport. We believe that the effect on aeromedical transfers will last until the very end of the COVID-19 pandemic. Even a few cases in a remote location with limited health care resources can increase demand for aeromedical transfers substantially. More than ever, it is imperative that clear plans are made between parties involved in patient transfer and that COVID-19 cases, either positive or suspected, are identified correctly, thereby optimizing resources and reducing risk to accompanying personnel.

## References

[1] Martin DT. Fixed wing patient air transport during the Covid-19 pandemic. Air Med J 2020;39(3):149–153. doi: 10.1016/j.amj.2020.04.001. 32540100 PMC7134971

[2] Teichman PG. International aeromedical evacuation. N Engl J Med 2007:356:262–270. 17229953 10.1056/NEJMra063651

[3] Poston JT, Patel BK, Davis AM. Management of critically ill adults with COVID-19. JAMA. 2020. doi: 10.1001/jama.2020.4914. 32215647

[4] Tran K, Cimon K, Severn M, Pessoa-Silva CL, Conly J. Aerosol generating procedures and risk of transmission of acute respiratory infections to healthcare workers: a systematic review. PLoS One 2012;7(4):e35797. 22563403 10.1371/journal.pone.0035797 PMC3338532

[5] Munshi L, Del Sorbo L, Adhikari NKJ, Prone position for acute respiratory distress syndrome. A systematic review and meta-analysis. Ann Am Thorac Soc 2017;14(Suppl 4):S280–S288. 29068269 10.1513/AnnalsATS.201704-343OT

